# From Phytochemical Characterization to Energy Metabolism-Driven Molecular Responses: The Anticancer Potential of *Lactarius deliciosus* (L.) Gray in Breast Cancer Cells

**DOI:** 10.3390/nu18061008

**Published:** 2026-03-23

**Authors:** Levent Gülüm, Emrah Güler, Emir Çapkınoğlu, Ayşe Büşranur Çelik, Yusuf Tutar

**Affiliations:** 1 Department of Plant and Animal Production, Mudurnu Süreyya Astarcı Vocational College, Bolu Abant İzzet Baysal University, Bolu 14030, Türkiye; 2 Medicinal and Aromatic Plants Research Group, Innovative Food Technologies Development Application and Research Center, Bolu Abant Izzet Baysal University, Bolu 14030, Türkiye; 3 Department of Horticulture, Faculty of Agriculture, Bolu Abant İzzet Baysal University, Bolu 14030, Türkiye; 4 Horticulture and Post-Harvest Research Group, Innovative Food Technologies Development Application and Research Center, Bolu Abant Izzet Baysal University, Bolu 14030, Türkiye; 5 Department of General Surgery, School of Medicine, Acibadem Mehmet Ali Aydinlar University, Istanbul 34638, Türkiye; 6 Department of Basic Medical Sciences, Faculty of Medicine, Division of Biochemistry, Recep Tayyip Erdogan University, Rize 53020, Türkiye; 7 Molecular Oncology Program, Health Sciences Institute, Recep Tayyip Erdogan University, Rize 53100, Türkiye; 8 Molecular Medicine Program, Health Sciences Institute, Recep Tayyip Erdogan University, Rize 53100, Türkiye; 9 Rize Training and Research Hospital, Recep Tayyip Erdogan University, Rize 53020, Türkiye; 10 Department of Basic Pharmaceutical Sciences, Faculty of Pharmacy, Division of Biochemistry, University of Health Sciences, Istanbul 34668, Türkiye

**Keywords:** mushroom extract, breast cancer cell MCF-7, apoptosis signaling, energy metabolism, phytochemical composition, anticancer agents

## Abstract

**Background/Objectives**: This study aimed to investigate the phytochemical composition, antioxidant capacity, and anticancer potential of methanol and ethanol extracts of *Lactarius deliciosus* (L.) Gray in MCF-7 breast cancer cells, focusing on their effects on energy metabolism and related molecular mechanisms. **Methods:** In *L. deliciosus* samples, total antioxidant activity and total phenolic content were determined spectrophotometrically, while individual phenolics were classified by HPLC and volatile aromatic compounds (VOCs) were determined by GC-MS. The anticancer effects of *L. deliciosus* in MCF-7 breast cancer were determined using RT-qPCR with 46 different genes. **Results:** Phytochemical profiling via HPLC and GC–MS revealed a rich diversity of bioactive compounds, including significant levels of gallic acid (298.89 µg/g), vanillic acid (191.98 µg/g), and succinic acid (724.73 µg/g). The extracts exhibited robust antioxidant activity and dose-dependent cytotoxicity, reducing cell viability to as low as 5.60% after 72 h. Molecular analysis through Reactome pathway enrichment and expression profiling of 46 genes demonstrated that *L. deliciosus* drives cancer cells into a metabolic impasse by reversing the Warburg effect. Key findings include the significant downregulation of glycolytic genes like *SLC2A1*/*GLUT1* (−12.34) and *HK2* (−1.71), alongside the repression of mitochondrial TCA cycle regulators such as *IDH1* (−17.81) and *OGDH* (−2.54). This metabolic collapse triggered G0/G1 phase cell cycle arrest and induced apoptosis. **Conlusions**: These results align with international benchmarks for *Lactarius* species while providing novel insights into the metabolic reprogramming mechanism. The results obtained in this study highlight that *L. deliciosus* emerges as a promising natural agent for therapeutic strategies targeting cancer bioenergetics.

## 1. Introduction

Phytochemicals, including flavonoids, terpenes, isothiocyanates, and polyphenols, play a critical role in disrupting the normal cell cycle and inducing apoptotic pathways to inhibit cancer development [1,2]. Compounds like chlorogenic acid and cinnamaldehyde have been reported to inhibit metastatic traits and downregulate the Akt signaling pathway, leading to apoptosis in breast cancer cells [3]. Dietary polyphenols and phytochemicals such as sulforaphane can modulate various signaling pathways involved in carcinogenesis and even reverse epigenetic alterations associated with cancer progression [4,5]. These natural agents, often working synergistically with conventional chemotherapeutics, represent a promising strategy to enhance efficacy and overcome drug resistance [6,7].

Edible mushrooms are increasingly valued for their rich array of bioactive compounds, such as polysaccharides, phenolics, and ergosterols, which contribute to potent antioxidant and anticancer activities [8,9]. Among these, *Lactarius deliciosus* stands out due to its significant nutritional value and high antioxidant activity, attributed to its rich polyphenol content [10,11]. Recent evidence substantiates the anticancer potential of *L. deliciosus*, with studies indicating that its polysaccharides can enhance immune responses and significantly suppress the proliferation of various cancer cell lines, such as HeLa and MCF-7, through the induction of apoptosis and cell cycle progression [12].

Energy metabolism-driven molecular responses in breast cancer cells represent a multifaceted and dynamically regulated network linked to oncogenic signaling and the tumor microenvironment [13,14,15]. The Warburg effect, mitochondrial *OXPHOS* repression, lipid metabolic reprogramming, and TCA cycle alterations collectively define the metabolic landscape of breast cancer in a subtype-specific manner [16,17]. Key molecular regulators including β-F1-ATPase, IF1, AMPK, GCN2-ATF4, and various RNA-binding proteins orchestrate these metabolic shifts at the post-transcriptional level [18,19]. Multi-omics integration has revealed the complexity of these responses, identifying clinically relevant biomarkers and therapeutic targets that hold promise for personalized strategies [20,21].

While breast cancer is marked by these significant metabolic changes, the specific molecular mechanisms by which edible mushrooms influence cancer cell energy metabolism remain poorly understood. Previous research on *L. deliciosus* has primarily concentrated on general cytotoxicity and antioxidant properties, often lacking detailed insights into metabolic signaling [12,22]. This study aims to clarify the anticancer potential of *L. deliciosus* in breast cancer cells by focusing on energy metabolism through the integration of phytochemical profiling (HPLC and SPME–GC–MS) with the expression profiling of 46 genes related to mitochondrial function and apoptosis, thereby providing a mechanistic framework and filling a critical gap in the literature regarding its potential as a functional food-derived anticancer agent.

## 2. Materials and Methods

### 2.1. Biological Material and Extract Preparation

#### Mushroom Source, Cultivation Conditions, and Extraction Approach

Fresh fruiting bodies of *L. deliciosus* (L.) Gray were obtained from a local market in Türkiye during the natural harvesting season. The identification was provided by Prof. Dr. Fuat Bozok (Figure S1). A voucher number, L. Gülüm-1001, was assigned to this material and is now preserved in the biochemistry laboratory of the Horticulture Department at Bolu Abant İzzet Baysal University.

The dried and powdered mushroom material was extracted using aqueous organic solvents, specifically 80% ethanol and 80% methanol, following a previously described protocol with minor modifications [23]. In brief, the samples were incubated at room temperature and subjected to 15 min of sonication (HY Technologies, Cairo, Egypt) to enhance extraction efficiency. After sonication, the mixture was filtered and then centrifuged. The supernatants were evaporated under reduced pressure and stored at −20 °C until use. For subsequent analyses, the extracts were dissolved in dimethyl sulfoxide (DMSO) to prepare stock solutions.

### 2.2. Chemical Composition and Antioxidant Characterization

#### 2.2.1. Quantification of Total Bioactive Constituents

##### Determination of Total Phenolics, Flavonoids, and Anthocyanins

The total phenolic content (TPC) was measured using a microscale Folin–Ciocalteu-based spectrophotometric assay, which was adapted from previously published methods [24,25]. The procedure roughly consisted of 160 μL of Folin–Ciocalteu reagent (10% *v*/*v*) to 10 μL of mushroom extract and the addition of 30 μL of 7% Na_2_CO_3_ (*w*/*v*) after vortexing the mixture. Samples were incubated in the dark for 2 h and read using a microplate reader (Thermo Scientific™ Multiskan™, Waltham, MA, USA) at 760 nm wavelength. Quantification was carried out using a gallic acid calibration curve prepared with the identical method and the results were expressed as gallic acid equivalents (GAEs) per gram of dry weight.

Total flavonoid content (TFC) was determined using a modified aluminum chloride-based colorimetric assay previously used by [26]. In this method, the extracts were sequentially reacted with sodium nitrite, aluminum chloride, and sodium hydroxide, and the absorbance was measured at 430 nm. Quantification was performed using a quercetin calibration curve, and the results were expressed as millimoles of quercetin equivalents per gram of dry weight.

Total anthocyanin content was assessed using an acidified ethanol method [27]. Samples were mixed with 30% ethanol containing 1% hydrochloric acid and analyzed spectrophotometrically at 540 nm. Anthocyanin levels were calculated as malvidin-3-glucoside equivalents per gram of dry weight using a specific equation.

Total anthocyanins = 16.7 × A_540_ × Df

where Df represents the dilution factor.

#### 2.2.2. Determination of Macronutrient Content

##### Soluble Proteins and Total Carbohydrates Assays

Soluble protein content was measured using a modified Bradford assay, while total carbohydrate content was determined using the phenol–sulfuric acid method previously performed by [27]. The protein content was quantified using the equation obtained from the known amount of bovine serum albumin at 595 nm and the carbohydrates content was determined by the known amount of glucose standards at 490 nm.

#### 2.2.3. Antioxidant Capacity Assessment

##### DPPH, ABTS, FRAP, and CUPRAC Methods

The antioxidant capacity was evaluated using four complementary spectrophotometric assays, with results expressed as millimolar (mM) ascorbic acid equivalents. All antioxidant assays were performed with 10 µL of sample and 190 µL of reagents. The ascorbic acid standards were prepared by a serial dilution of 2 mM ascorbic acid stock and went through the same procedure within each assay.

DPPH Radical Scavenging Activity: The DPPH scavenging activity was determined by reacting samples with DPPH dissolved in ethanol, adjusted to an initial absorbance of 0.70–0.80 at 517 nm. The mixture was then incubated for 15 min at room temperature, followed by measurement of the absorbance at the same wavelength [28].

CUPRAC Activity: The activity was assessed through a reaction containing 10 mM Cu^2+^, 7.5 mM neocuproine, and 1 M ammonium acetate buffer. The sample–reagent mixtures were incubated for 20 min at room temperature, and the absorbance was recorded at 450 nm [29].

ABTS^+^ Scavenging Capacity: The assay was performed using pre-formed ABTS radicals (7 mM ABTS with potassium persulfate), which were diluted to an absorbance of 0.700. After dilution, the extract was reacted for 15 min and read at 734 nm [30].

Ferric Reducing Antioxidant Power (FRAP): FRAP reagent was prepared by the protocol proposed by [31]. The absorbance was measured at 593 nm after incubation under ambient conditions.

### 2.3. Targeted Phytochemical Profiling in L. deliciosus

#### HPLC-Based Phenolic Profiling

Phenolic profiling was performed using high-performance liquid chromatography (HPLC), following a protocol adapted from [32]. The analyses utilized a Waters HPLC system (Milford, MA, USA) equipped with gradient pumps, an autosampler injector (20 µL loop), and UV–fluorescence detection. Chromatographic separation was achieved on a reversed-phase C18 column (150 × 3.9 mm, 4 µm), protected by a guard column. The elution process was conducted at a flow rate of 1.0 mL/min, employing a binary gradient consisting of solvent A (methanol/acetic acid/water, 10:2:88, *v*/*v*) and solvent B (methanol/acetic acid/water, 90:2:8, *v*/*v*). Detection occurred at 280 nm, while fluorescence signals were monitored with excitation/emission wavelengths of 278/360 nm and 330/374 nm. Peak homogeneity was confirmed through diode array spectral analysis, with acceptance criteria set at ≥99.5% spectral similarity. For the quantitative determination, a broad range of phenolic standards was dissolved in methanol and serially diluted from an initial concentration of 80 µg/mL. All standard and sample solutions were filtered through 0.45 µm cellulose acetate membranes before analysis. An internal standard approach utilized 2,5-dihydroxybenzaldehyde (34.4 µg/mL) to enhance analytical accuracy. Compound identification was based on matching retention times and spectral characteristics with reference standards, and calibration curves were generated using linear regression (R^2^ > 0.99). Final phenolic concentrations were calculated from their corresponding regression equations and expressed accordingly.

### 2.4. Volatile Metabolite Analysis

#### SPME–GC–MS-Based Aroma Characterization

Volatile metabolite analysis was conducted by modifying the SPME–GC–MS protocol described by [33]. The analyses were carried out using a Shimadzu GC–MS-QP2010 system (Guangzhou, Guangdong, China), which was equipped with an Rxi-5ms fused-silica capillary column (60 m × 0.25 mm i.d., 0.25 µm film thickness). In brief, approximately 0.9 g of finely ground and homogenized sample was placed in a 20 mL sealed headspace vial and equilibrated at 70 °C for 30 min. The headspace volatiles were then adsorbed using a 75 µm CAR/PDMS SPME fiber and thermally desorbed in the GC injector at 250 °C under splitless conditions. Chromatographic separation was achieved using helium as the carrier gas at a flow rate of 1.0 mL/min. A multi-step oven temperature program was followed, starting at 45 °C and gradually increasing to a final temperature of 240 °C, with intermediate holding and ramping stages to ensure efficient resolution of the volatile compounds. Mass spectrometric detection was performed in electron impact (EI) mode at 70 eV, scanning ions in the m/z range of 30–650. The temperatures of the interface, ion source, and quadrupole were maintained at 250 °C, 230 °C, and 150 °C, respectively. Volatile constituents were identified by matching mass spectra and calculated retention indices with those of reference standards and spectral libraries (FFNSC 1.2 and Wiley 7), using a C7–C20 n-alkane series for index calculation. The relative abundances of the volatile compounds were expressed as percentage peak area ratios, calculated by dividing the individual peak area by the total chromatographic area.

### 2.5. Cell-Based Bioactivity Assessment

#### Cell Culture Conditions

Cell-based assays were conducted by slightly modifying the protocol established by [34]. Human MCF-7 breast adenocarcinoma cells and the human normal skin fibroblast cell line CCD-1072Sk (ATCC^®^) were maintained in Dulbecco’s modified eagle medium (DMEM) (EuroClone, Via Figino, Pero, Italy), supplemented with 10% fetal bovine serum and 1% glutamine. The cells were cultured at 37 °C in a humidified environment with 5% CO_2_. When the cells reached 80–90% confluence, they were subcultured using 0.25% trypsin–EDTA and plated in 96-well plates at densities ranging from 5 × 10^3^ to 1 × 10^4^ cells per well. 5-Fluorouracil (5-FU), a widely used chemotherapeutic drug in cancer therapy, was employed as a positive control to evaluate cytotoxic effects. MCF-7 cells were treated with varying concentrations of 5-FU, and cell viability was assessed under the same experimental conditions used for *L. deliciosus* extracts.

After allowing the cells to attach, they were exposed to serially diluted concentrations of *L. deliciosus* extracts (up to 1000 µg/mL) for 48 and 72 h to assess cytotoxic activity. For control groups, cells were treated with equivalent concentrations of the extraction solvent. Each experiment was performed independently in duplicate, with at least four technical replicates per treatment. Cell viability was evaluated using the MTT assay, with absorbance readings taken at 570 nm (Thermo Scientific, Multiskan Go, Vantaa, Finland). Half-maximal inhibitory concentration (IC_50_) values were calculated using GraphPad Prism software (version 8.0.2).

### 2.6. Flow Cytometry-Based Cellular Responses

#### 2.6.1. Cell Cycle Distribution Analysis

Cell cycle progression was assessed using the Sigma-Aldrich MAK344 Cell Cycle Analysis Kit (Sigma-Aldrich, St. Louis, MO, USA), following the manufacturer’s instructions. MCF-7 cells were exposed to IC_50_ concentrations of the extracts for 96 h. The cells were then collected, washed with phosphate-buffered saline (PBS), and fixed in ice-cold 70% ethanol. After fixation, the cells were washed with the assay buffer and incubated with a staining mixture containing Rnase (enzyme A) and a DNA-binding fluorescent dye. Following incubation in the dark, the samples were analyzed using flow cytometry (Beckman Coulter Cytoflex, Indianapolis, IN, USA) to determine the distribution of cells across different phases of the cell cycle. All measurements were performed in triplicate to ensure analytical reliability. Cell cycle distribution was evaluated by propidium iodide (PI) staining following ethanol fixation and analyzed by flow cytometry [35].

#### 2.6.2. Apoptosis Detection (Annexin V-FITC/PI Staining)

Apoptotic responses were evaluated using the ApopNexin™ FITC Annexin V/PI Apoptosis Detection Kit (Merck, Darmstadt, Germany). MCF-7 cells, treated with IC_50_ concentrations for 96 h, were harvested, washed, and resuspended in binding buffer. They were then stained with FITC-conjugated Annexin V and propidium iodide (PI). After a 15 min incubation at room temperature in the absence of light, the samples were analyzed by flow cytometry. This method allowed for the differentiation between viable, early apoptotic, late apoptotic, and necrotic cell populations [36].

### 2.7. Gene Expression and Metabolic Pathway Analysis

#### RT-qPCR-Based Profiling and Reactome Analysis

Gene expression profiles from MCF-7 cells treated with methanolic extracts of *L. deliciosus* were analyzed to understand the changes in cancer-related metabolic and signaling pathways. Differentially expressed genes were mapped to the Reactome database to identify significantly enriched biological pathways, focusing on interactions with a *p*-value of ≤0.05. Pathway enrichment analysis was conducted using a hypergeometric test, with multiple comparisons adjusted via the Benjamini–Hochberg procedure. A false discovery rate (FDR) threshold was set at 0.05 to ensure statistical reliability.

For gene expression analysis, total RNA was isolated using a commercial silica membrane-based RNA extraction kit according to the manufacturer’s protocol. RNA concentration and purity were initially evaluated spectrophotometrically (Eppendorf BioSpectrometer^®^, Hamburg, Germany) by measuring A260/A280 ratios, and samples with ratios between 1.9 and 2.1 were considered suitable for downstream analysis. Complementary DNA (cDNA) was synthesized from purified RNA using a reverse transcription kit following the manufacturer’s instructions. Quantitative real-time PCR (RT-qPCR) was performed using SYBR Green (EuroClone, Via Figino, Pero, Italy) chemistry on a real-time PCR detection system. Primer sequences used for each target gene are provided in Supplementary Table S1.

Each experiment was conducted with three independent biological replicates, and each reaction was performed in technical triplicate to ensure analytical reproducibility. Amplification efficiency of primer pairs was evaluated using standard curve analysis and was within acceptable limits (90–110%). GAPDH was used as the internal reference gene due to its stable expression across experimental conditions. Relative gene expression levels were calculated using the 2^−ΔΔCt^ method. The resulting expression patterns were integrated with the pathway enrichment results to provide mechanistic insights into the molecular responses induced by *L. deliciosus* treatment.

A targeted panel of 46 genes involved in mitochondrial function, metabolic regulation, and apoptosis was selected based on the previous literature, and gene expression levels were normalized using GAPDH as the reference gene.

### 2.8. Statistical Analysis

The extraction experiments were conducted according to randomized plots design with three independent replicates and evaluated using JMP Pro 16 software (SAS, Cary, NC, USA). Data were first tested for variance homogeneity using Levene’s test, followed by one-way ANOVA to determine the effects of extraction solvent. Mean comparisons were performed using Fisher’s Least Significant Difference (LSD) test at a confidence level of 95% (α = 0.05). Analyses related to cell viability and IC_50_ calculations were carried out using GraphPad Prism software (version 8.0.2).

## 3. Results

### 3.1. Quantification of Total Bioactive Constituents

#### Total Phenolics, Flavonoids, and Anthocyanins

The total phenolic, flavonoid, and anthocyanin contents of extracts from *L. deliciosus* significantly differed based on the solvent used (Table 1). The methanol extract had a significantly higher TPC of 6.54 mM compared to the ethanol extract, that of 3.50 mM (*p* < 0.05). In contrast, the TFC and the total anthocyanin content were higher in ethanol extract, with TFC values of 2.72 mM and anthocyanin values of 1021.80 mM, compared to 2.05 mM TFC and 682.97 mM anthocyanins in the methanol extract (*p* < 0.05).

### 3.2. Macronutrient Profiling

#### Soluble Proteins and Total Carbohydrates

The total protein and carbohydrate contents of *L. deliciosus* extracts significantly changed based on the solvent used (Table 2). The methanol extract demonstrated a statistically higher total protein content (24.52%) compared to the ethanol extract (9.67%) (*p* < 0.05). In contrast, no significant difference was observed between the ethanol (34.45%) and methanol (37.14%) extracts regarding total carbohydrate content (*p* > 0.05).

### 3.3. Antioxidant Capacity of L. Deliciosus

#### DPPH, ABTS, FRAP, and CUPRAC Activities

The total antioxidant capacities of *L. deliciosus* extracts varied significantly based on the solvent used (Table 3). The methanol extract possessed stronger activity in terms of free radical scavenging and reduction capacity, with notably higher values compared to the ethanol extract in the DPPH assay (2.91 mM) and particularly in the CUPRAC assay (35.88 mM) (*p* < 0.05). This result aligns with the high levels of redox-active phenolic compounds, such as gallic acid, vanillic acid, and chlorogenic acid, found in the methanol extract. Conversely, the ethanol extract showed significantly greater antioxidant capacity than the methanol extract in the ABTS (15.95 mM) and the FRAP assays (14.40 mM) (*p* < 0.05).

### 3.4. Targeted Phytochemicals by HPLC and GC-MS

#### Phenolics Diversity in *L. deliciosus* Extracts

HPLC analysis revealed that the phenolic and organic acid composition of *L. deliciosus* is significantly influenced by the extraction solvent used. The methanol extract showed a more diverse profile, particularly regarding phenolic acids. High levels of gallic acid (298.89 µg/g), vanillic acid (191.98 µg/g), chlorogenic acid (87.30 µg/g), caffeic acid (76.20 µg/g), and syringic acid (70.26 µg/g) were detected. Additionally, quercitrin (8.91 µg/g), which was identified only in the methanol extract, further exemplifies the solvent sensitivity of the flavonoid content. Given the antioxidant and metabolic regulatory effects of these compounds, the methanol extract is considered to have greater potential for biological activity. In contrast, the ethanol extract demonstrated a dominant profile, particularly with certain phenolics and organic acids. Notably, (−)-epicatechin (561.30 µg/g) and succinic acid (724.73 µg/g) were detected only in the ethanol extract at high concentrations. Moreover, significant amounts of vanillic acid (209.19 µg/g), syringin hydrate (145.46 µg/g), procatechin (84.59 µg/g), and chlorogenic acid (59.79 µg/g) were identified in ethanolic extract (Table 4). The presence of succinic acid, a metabolite directly associated with the TCA cycle, aligns with the biological effects of *L. deliciosus* on energy metabolism.

### 3.5. VOCs in L. deliciosuss

SPME–GC–MS analysis indicated that the aromatic profile of the *L. deliciosus* specimen is primarily composed of organic acids and oxygenated volatile compounds. Organic acids represent 52.55% of the total volatile compounds, making them the dominant group. Within this category, acetic acid (30.02%) and capric acid (21.81%) significantly contribute to the mushroom’s sharp, acidic, and characteristic aroma. Aldehydes account for 9.91% of the aroma profile, with the presence of hexanal and benzaldehyde. Compounds from the ketone group make up 4.26%, with 2-heptanone and 1-octen-3-one noted as contributors to mushroomy and slightly metallic aromas. Alcohols form the second most dominant fraction of the volatile profile, totaling 14.94%. Notably, 1-octen-3-ol (10.00%) and 3-octanol (4.94%) are the primary contributors to the mushroom’s characteristic earthy and mushroom-like aroma. Lactone derivatives (4.81%) and furans (2.00%) add sweet, caramel-like, and roasted aromatic nuances. Nitrogen-containing heterocyclic compounds represent 4.83% of the profile, with 2-acetylpyrrole (3.81%) and methylpyrazine (1.02%) contributing nutty and roasted notes. Additionally, limonene (3.92%) imparts a citrusy and refreshing terpene character, while alkanes and aromatic/polycyclic compounds are present in smaller amounts (Table 5). These results suggest that *L. deliciosus* possesses a complex and multi-component aroma profile, with a significant emphasis on mushroom-specific alcohols and organic acids.

### 3.6. In Vitro Cell-Based Bioactivity Influenced by L. deliciosus Treatment

**Figure 1:**
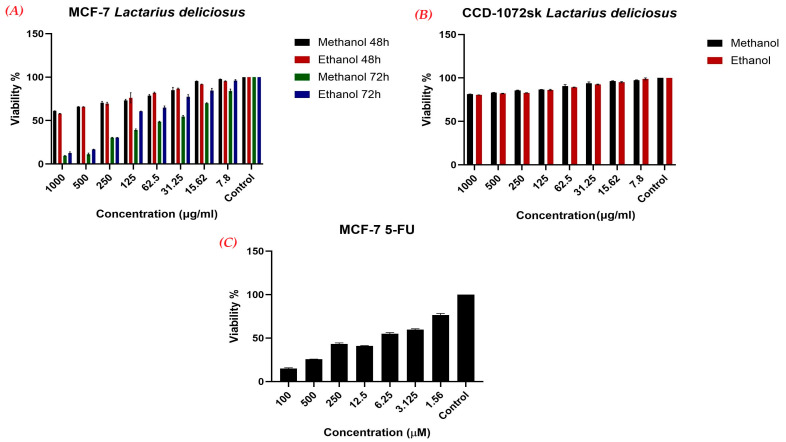
Cell viability ratios in MCF-7 (**A**) and CCD-1072sk (**B**) cell lines in response to *L. deliciousus* treatment, as well as the effect of 5-FU anticancer drug on MCF-7 breast cancer cells (**C**).

The MTT analysis demonstrated that both methanol and ethanol extracts of *L. deliciosus* exerted significant dose- and time-dependent cytotoxic effects on MCF-7 cells, with a sharp decline in viability observed at higher concentrations (250 µg/mL to 1000 µg/mL) and prolonged exposure. While 1000 µg/mL of the methanol extract resulted in 61% cell viability after 48 h, it dramatically reduced survival to 9.53% after 72 h; similarly, the ethanol extract at this dose reached a viability of 57.94% at 48 h and a notable 5.60% at 72 h. A strong dose-dependent pattern was evident at the 72 h mark, where six of the seven methanol concentrations (7.8–1000 µg/mL) reduced viability below 50%, while the ethanol extract exhibited slightly superior efficacy at this time point, bringing viability down to approximately 15% to 20% at 250 µg/mL. In contrast, lower concentrations ranging from 15.62 to 62.5 µg/mL maintained relatively high viability rates, with values around 95% to 100% at 7.8 µg/mL and in control groups (Figure 1A). In the healthy cells, the effect of ethanol extract of *L. deliciosus* on cell viability ranged from 80.55% to 98.92% in 1000 µg/mL to 7.8 µg/mL, respectively. Similarly, methanolic extract resulted in cell viabilities of 81.37% to 97.45 depending on the decreasing doses (Figure 1B). The anticancer drug caused a cell viability of 76.44% in a 1.56 µm dose, while its effect rose up to 15.07% at a 100 µm dose (Figure 1C). These findings indicate that *L. deliciosus* extracts, particularly the ethanol fraction and prolonged exposure times, significantly inhibit MCF-7 cell proliferation and lead to a marked decrease in cell viability.

### 3.7. Flow Cytometry-Based Cellular Responses

#### 3.7.1. Cell Cycle Changes in Response to *L. deliciosus* Treatment

**Figure 2:**
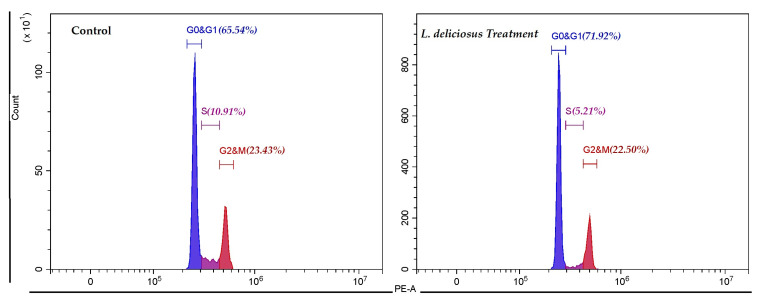
Cell cycle alteration in the control and *L. deliciosus*-treated MCF-7 breast cancer cells.

Cell cycle analysis using PI staining revealed that the application of *L. deliciosus* causes a significant arrest in the G0/G1 phase in MCF-7 breast cancer cells. In the untreated (control) group, 65.54% of the cells were in the G0/G1 phase, 10.91% in the S phase, and 23.43% in the G2/M phase. The IC_50_ dose of *L. deliciosus* resulted in an increase in the G0/G1 phase population to 71.92%, while the percentage of cells in the S phase decreased by nearly half to 5.21%. The proportion of cells in the G2/M phase remained similar to that of the control group, at 22.50% (Figure 2).

#### 3.7.2. Apoptosis Induction by *L. deliciosus*

**Figure 3:**
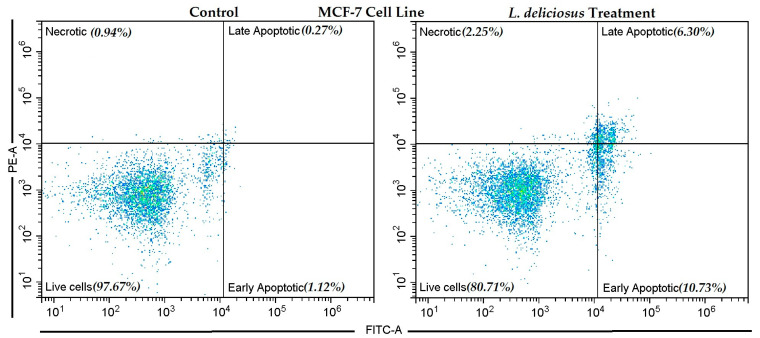
The induction of apoptosis in the control and *L. deliciosus*-treated MCF-7 breast cancer cells.

*L. deliciosus* exhibited a significant apoptotic effect on MCF-7 breast cancer cells in the Annexin V–FITC/PI flow cytometry assay. In the control group, the vast majority of cells were viable, with 97.67% showing viability. The rates of early apoptotic, late apoptotic, and necrotic cells were very low, at 1.12%, 0.27%, and 0.94%, respectively. The treatment with *L. deliciosus* led to a significant decrease in viable cells, which dropped to 80.71%. The rate of early apoptotic cells increased roughly tenfold to 10.73%, while the rate of late apoptotic cells rose to 6.30%. Additionally, the increase in necrotic cells to 2.25% suggests that the treatment also induced a limited level of necrosis (Figure 3).

### 3.8. Genetic Modification and Regulation of Metabolic Pathwas

#### 3.8.1. RT-qPCR-Based Profiling and Reactome Analysis

Significant changes in gene expression related to energy metabolism, lipid metabolism, and biosynthetic pathways were observed in MCF-7 breast cancer cells treated with a methanol extract of the *L. deliciosus* mushroom. Several genes were upregulated, including *BCAT1* (+13.88), which is involved in amino acid metabolism and metabolic stress adaptation; *G6PD* (+12.95), a key enzyme in the pentose phosphate pathway; *SLC2A2* (+8.37), which is responsible for glucose transport; *PFKFB4* (+2.14), a regulatory component of glycolysis; and *CPT1C* (+2.26), associated with mitochondrial fatty acid transport. Additionally, increases in the *PHGDH* (+1.47), *PSPH* (+1.32), and *TKTL1* (+1.65) genes, which are linked to serine biosynthesis and metabolic reprogramming, indicate that the cells have developed an adaptive response to metabolic stress.

**Figure 4:**
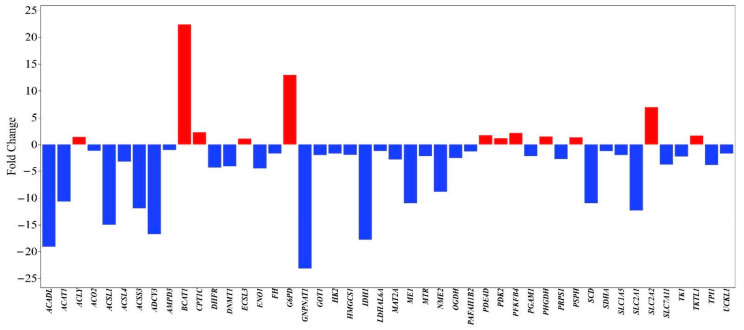
Gene expression profile of MCF-7 breast cancer cells induced by *the L. deliciosus* treatment.

In contrast, many genes associated with glycolysis, mitochondrial energy production, and lipid biosynthesis were significantly downregulated. The genes *SLC2A1* (−12.34), *HK2* (−1.71), *ENO1* (−4.48), *PGAM1* (−2.15), and *TPI1* (−9.61), which are involved in glucose uptake and glycolysis, showed decreased expression. Significant reductions were also noted in the genes *IDH1* (−17.81), *OGDH* (−2.54), *SDHA* (−1.25), *FH* (−1.68), and *ACO2* (−1.19), which are associated with the mitochondrial TCA cycle and oxidative metabolism. Regarding lipid metabolism, the downregulation of the ACADL (−19.09), *ACAT1* (−14.67), *ACSL1* (−11.92), *ACSL4* (−3.19), *ACSS3* (−11.92), and *SCD* (−10.45) genes indicates severely limited fatty acid oxidation and biosynthesis. Furthermore, the reduction in the expression of the *DNMT1* (−4.10), *DHFR* (−4.30), *PRPS1* (−2.72), and *TK1* (−2.24) genes, which are linked to nucleotide synthesis and epigenetic regulation, suggests a weakened capacity for cellular proliferation (Figure 4). This gene expression profile may suggest that the *L. deliciosus* extract exerts an anticancer effect on breast cancer cells by inducing metabolic reprogramming, which involves the suppression of glycolysis, mitochondrial energy production, and lipid metabolism.

#### 3.8.2. Metabolic Pathway Enrichment Analysis Findings

The Reactome pathway analysis of MCF-7 cells treated with *L. deliciosus* extract revealed a pronounced repression profile in general metabolism (FDR = 2.64 × 10^−14^), specifically targeting the citric acid cycle (FDR = 6.12 × 10^−6^), aerobic respiration, and the electron transport chain (FDR = 1.81 × 10^−5^). Transcriptomic data showed significant downregulation of glucose transport via *SLC2A1*, hexose transport (FDR = 3.27 × 10^−2^), and pyruvate metabolism, alongside structural compromises in mitochondrial Fe–S cluster biogenesis, Complex III assembly, and protein import. Lipid metabolism was severely restricted through the suppression of fatty acid acyl–CoA biosynthesis (FDR = 4.0 × 10^−5^), *SREBP*-mediated cholesterol regulation, and beta-oxidation, while amino acid utilization was curtailed via the inhibition of BCAA catabolism and SLC-mediated transport. Furthermore, the extract suppressed the mitotic G1/S transition (FDR = 1.87 × 10^−2^), nucleotide salvage, and translational machinery—including the 43S ribosomal complex—while simultaneously inhibiting epigenetic regulators such as WDR5 (FDR = 1.05 × 10^−3^) and DNA methylation pathways. Conversely, a distinct positive enrichment was observed in *NFE2L2* (*NRF2*)-mediated pathways (FDR < 10^−5^), specifically involving the regulation of the pentose phosphate pathway and antioxidant response elements.

## 4. Discussion

The phytochemical characterization of *L. deliciosus* in this study reveals a rich and diverse profile that aligns with, and in some aspects surpasses, previously reported values for this species. Our findings regarding TPC show that the methanol extract (6.54 mM) yielded significantly higher phenolic concentrations compared to the ethanol extract (3.50 mM). This observation is consistent with the findings of [37], who reported that methanol is the most effective solvent for recovering phenolics from Ordu-sourced *L. deliciosus.* However, the absolute concentrations vary across geographies; while samples from China reached up to 13.68 mg GAE/g in aqueous fractions [38], our samples demonstrate a competitive phenolic density that underpins their biological activity. The presence of specific flavonoids and anthocyanins in our ethanol extracts further suggests that solvent polarity plays a critical role in the differential extraction of these secondary metabolites.

The individual phenolic acid profile identified by HPLC in our study shows distinct geographical variations when compared to European and Asian samples. We identified gallic acid (298.89 μg/g) and vanillic acid (191.98 μg/g) as dominant components. In contrast, studies from Spain identified homogentisic acid as the primary phenolic acid [39], while research from Poland and Portugal frequently highlighted p-hydroxybenzoic acid [40]. Interestingly, the detection of procatechin (84.59 μg/g) in our samples aligns with the Spanish profile, where catechin derivatives were also noted. These discrepancies in dominant phenolic acids, such as the prevalence of pyrogallol in some Turkish studies [37] versus gallic acid in ours, confirm that the chemical plasticity of *L. deliciosus* is highly sensitive to regional environmental factors and soil composition.

Regarding antioxidant potential, our extracts demonstrated robust radical scavenging activity, with the methanol extract showing a DPPH value of 2.91 mM and a CUPRAC value of 35.88 mM. This strong antioxidant performance is directly linked to the high concentrations of redox-active compounds like gallic and chlorogenic acids found in our samples. When compared to international benchmarks, where IC_50_ values for DPPH range from 4.07 mg/mL (Ordu) to 17 mg/mL (Kastamonu), our results indicate a highly efficient antioxidant system. Furthermore, the substantial lipid peroxidation inhibition observed in our study is consistent with the protective roles described in Spanish and Portuguese *L. deliciosus* samples [39], emphasizing the species’ global consistency as a functional food source with high oxidative stress mitigation potential. Previous research shows that secondary metabolite profiles vary by geographic origin, and substantial differences were reported in metabolite composition among *Schisandra chinensis* populations in China, which are linked to their origin and growing conditions [41]. Similar variations in rice metabolomes have been associated with specific genetic loci, indicating that geographic factors influence metabolite diversity [42]. Other studies, such as those on *Artemisia* spp. in Cameroon [43] and *Vaccinium macrocarpon* across North American coastal regions [44], also highlight geography-linked phytochemical diversity. In the light of the literature, the compositional differences in *L. deliciosus* in this study and the previous study could be attributed to ecological differences.

A unique aspect of our study is the integration of nutritional composition with metabolic signaling markers. We found significant levels of protein (24.52%) and carbohydrates (37.14%) in our *L. deliciosus* samples. While these values are more modest than the exceptionally high protein levels reported in some Turkish samples [37], they exceed the carbohydrate-heavy profiles seen in certain Chinese varieties [38]. Crucially, the identification of succinic acid (724.73 μg/g) in our ethanol extract provides a novel phytochemical link to the energy metabolism responses we observed. This presence of TCA cycle intermediates suggests that the mushroom’s anticancer effect may be facilitated by its specific organic acid profile, offering a mechanistic explanation for the suppression of mitochondrial energy production in breast cancer cells.

The volatile organic compound (VOC) profile of *L. deliciosus* identified in our study reveals a complex chemical composition dominated by organic acids, alcohols, and aldehydes, which both aligns with and distinguishes itself from international benchmarks. Consistent with studies on Chinese *L. deliciosus* samples, organic acids were found to be the major chemical group [37], accounting for 52.55% of the total area in our analysis. Specifically, the high prevalence of acetic acid (30.02%) and capric acid (21.81%) in our samples reflects a broader trend within the *Lactarius* genus where acids and aldehydes frequently constitute the largest proportion of the volatile bouquet.

A hallmark of mushroom volatiles is the presence of eight-carbon (C8) compounds, often referred to as mushroom alcohol [45]. In our study, 1-octen-3-ol was a significant component (10.00% area), alongside 3-octanol (4.94%). This is in strong agreement with the volatile profiles of *L. deliciosus* from Transylvania and other European regions, where C8 alcohols and ketones are cited as primary contributors to the characteristic fungal aroma [46,47]. While [48] identified oxidized sesquiterpenes as character impact odorants in *L. hatsudake*, our profile for *L. deliciosus* emphasizes a higher concentration of aliphatic acids and alcohols, potentially due to the specific extraction methodologies or environmental factors associated with the collection site.

Furthermore, our GC–MS analysis detected unique heterocyclic and nitrogen-containing compounds, such as 2-acetylpyrrole (3.81%) and methylpyrazine (1.02%), which are often associated with complex aroma profiles and potential biological activities [49]. The presence of L-limonene (3.92%) and various aldehydes like benzaldehyde (1.61%) further enriches the chemical diversity of our samples compared to those reported in the literature. As noted by [50], drying methods significantly affect the preservation of esters and aromatic substances. Our detection of various aldehydes and ketones indicates that our optimized extraction successfully captured the volatile complexity of the mushroom. These results contribute to a deeper understanding of the *L. deliciosus* volatilome and provide a chemical basis for the metabolic reprogramming observed in our anticancer molecular assays.

Specific sesquiterpenes and fatty acid derivatives detected in our samples is consistent with the broad chemical defense strategies observed in the *Lactarius* genus. Studies on *L. hatsudake* identified oxidized sesquiterpenes such as isolongifolanone and alpha-cedrene epoxide as character impact odorants [48,51]. Similarly, research on *L. quetus* and *L. volemus* emphasized the role of azulene-type sesquiterpenes (lactarorufin A and B) as primary protective agents [47,52]. Our identification of diverse fatty acid methyl esters and volatile acids supports the notion that *L. deliciosus* utilizes these compounds not only for ecological defense but also as bioactive agents that may contribute to the metabolic reprogramming of cancer cells observed in our molecular assays.

The antiproliferative effect of *L. deliciosus* observed in our study is characterized by a strategic disruption of the cell cycle and the initiation of programmed cell death, aligning with key findings in fungal oncology. Our results demonstrate that *L. deliciosus* extracts induce significant cell cycle arrest in MCF-7 cells, a phenomenon also reported for other wild mushrooms such as *Suillus collinitus* and *Leccinum vulpinum* [53,54]. Specifically, the ability of fungal extracts to increase *p53* expression and trigger G1 or G2/M phase arrest [55], as documented in the northeast of Portugal samples, parallels the inhibitory patterns seen in our assays [53]. This suggests that the bioactive components in *L. deliciosus*, particularly its unique phenolic profile, act as potent modulators of cell cycle checkpoints in breast cancer models.

The induction of apoptosis is a hallmark of the anticancer potential of *Lactarius* species. Our molecular data, which indicate a transition toward apoptosis in MCF-7 cells, are strongly supported by recent studies on mushroom polysaccharides [56]. The novel heteropolysaccharides LDG-A and LDG-B have been shown to modulate macrophage activity and exhibit marked antitumor effects in vivo by influencing transcriptomic pathways related to cell survival [57,58]. Furthermore, while some research has focused on the protective effects of *L. deliciosus* polysaccharides against oxidative damage in PC12 cells [59], the overarching consensus in recent reviews is that these fungal metabolites can selectively switch from cytoprotective to pro-apoptotic roles in malignant environments [60].

A critical comparison can be made regarding the specificity of these effects across different cancer types. While our work focuses on breast cancer (MCF-7), *L. deliciosus* has also demonstrated significant anti-glioma potential, inducing apoptosis in U87MG and LN-18 glioblastoma cell lines [61]. This cross-cancer efficacy highlights the mushroom’s broad therapeutic window [26]. Additionally, toxicological analyses of *L. deliciosus* extracts as biopesticides have confirmed their safety profile in non-target organisms [62], further reinforcing the viability of its bioactive compounds for human pharmacological applications. By integrating our findings with these established models, we provide a more comprehensive understanding of how *L. deliciosus* effectively suppresses mitochondrial energy production while simultaneously activating the apoptotic machinery to eradicate cancer cells.

The collective alterations in these pathways indicate that *L. deliciosus* extract exerts a multifaceted anticancer effect by driving breast cancer cells into a metabolic impasse through the simultaneous suppression of mitochondrial bioenergetics, fatty acid synthesis, and glycolytic flux. The observed weakening of the Warburg effect, evidenced by the suppression of *GLUT1* and pyruvate metabolism, suggests a disruption of the fundamental energy preference of cancer cells [63]. While the activation of *NFE2L2*-mediated signaling reflects a compensatory survival response to mushroom-induced oxidative stress and a need for NADPH production via the pentose phosphate pathway [64], the severe repression of *SREBP* and epigenetic complexes like *WDR5* indicates a loss of the transcriptional and lipid-homeostatic control required for membrane biogenesis and rapid proliferation [65,66,67]. These findings suggest that the extract inhibits cancer growth by multifaceted targeting of metabolic networks, nutrient uptake, and protein synthesis, aligning with established metabolism-centered therapeutic strategies that lead to cell cycle arrest and apoptosis.

## 5. Conclusions

This study evaluates *L. deliciosus* extracts, highlighting their rich phytochemical profile and significant antioxidant capacity, which contribute to their strong antitumor effects on MCF-7 breast cancer cells. Our findings show that *L. deliciosus* treatment induces a metabolic stalemate in cancer cells by reversing the Warburg effect through the suppression of glycolysis, particularly by targeting *SLC2A1*/*GLUT1*, and disrupting mitochondrial TCA cycle genes like *IDH1*, *OGDH*, and *FH*. This metabolic collapse leads to G_0_/G_1_ phase cell cycle arrest and the induction of apoptosis, a mechanism supported by the presence of high levels of succinic and gallic acids identified in HPLC analysis. The primary strength of this research is its mechanistic depth, as it bridges the gap between phytochemical characterization and the expression profiling of 46 energy metabolism-related genes using Reactome pathway analysis. Furthermore, the comprehensive documentation of nutritional value, volatile profiles, and phenolic content offers a holistic view of the mushroom’s functional food potential. However, the study is limited by its focus on a single cell line, necessitating further comparative studies across diverse breast cancer subtypes. Additionally, while these in vitro results are promising, in vivo validation is required to assess the bioavailability and systemic efficacy of the observed metabolic modulation. Future investigations should also aim to isolate and test individual bioactive compounds to determine their specific contributions to the observed synergistic antitumor activity. In conclusion, *L. deliciosus* emerges as a compelling candidate for developing natural therapeutic strategies targeting cancer bioenergetics.

## Figures and Tables

**Table 1:** Total phenolics, flavonoids, and anthocyanin contents of *L. deliciosus* ethanol and methanol extracts (per gram).

Solvent	TPC (mM)	TFC (mM)	Tant (mM)
Ethanol	3.50 ± 0.15 ^b^	2.72 ± 0.11 ^a^	1021.80 ± 42.68 ^a^
Methanol	6.54 ± 0.27 ^a^	2.05 ± 0.09 ^b^	682.97 ± 28.53 ^b^

Different letters in the same column indicate significant differences at *p* < 0.05.

**Table 2:** Total protein and carbohydrate content of *L. deliciosus* extracts depending on the solvent.

Solvent	Protein (%)	Carbohydrates (%)
Ethanol	9.67 ± 0.40 ^b^	34.45 ± 1.44 ^a^
Methanol	24.52 ± 1.02 ^a^	37.14 ± 1.55 ^a^

Different letters in the same column indicate significant differences at *p* < 0.05.

**Table 3:** Total antioxidant activities of *L. deliciosus* extracts in different solvents.

Solvent	DPPH (mM)	CUPRAC (mM)	ABTS (mM)	FRAP (mM)
Ethanol	1.29 ± 0.05 ^b^	7.42 ± 0.31 ^b^	15.95 ± 0.67 ^a^	14.40 ± 0.60 ^a^
Methanol	2.91 ± 0.12 ^a^	35.88 ± 1.50 ^a^	12.91 ± 0.54 ^b^	7.76 ± 0.32 ^b^

Different letters in the same column indicate significant differences at *p* < 0.05.

**Table 4:** The phenolic compounds detected in *L. deliciosus* by HPLC.

Phenolic and Organic Acid Profiles	*L. deliciosus* Ethanol (µg/g)	*L. deliciosus* Methanol (µg/g)
Gallic Acid	14.09	298.89
4-Aminobenzoic Acid	3.80	n.d.
Procatechin	84.59	77.73
Chlorogenic Acid	59.79	87.30
Syringic Acid	n.d.	70.26
4-Hydroxybenzoic Acid	32.55	24.21
Syringin Hydrate	145.46	95.64
Caffeic Acid	20.19	76.20
Vanillic Acid	209.19	191.98
Ferulic Acid	4.43	n.d.
Synapic Acid	n.d.	n.d.
Coumaric Acid	n.d.	n.d.
Rutintrihydrate	n.d.	n.d.
Quercitrin	n.d.	8.91
(−)-Epicatechin	561.30	n.d.
(+)-Catechin	n.d.	n.d.
Salicylic Acid	n.d.	n.d.
Succinic Acid	724.73	99.76

n.d.—not detected.

**Table 5:** SPME–GC–MS profile of *L. deliciosus*.

Group	Compound Name	% Area	RI
1. Organic Acids	Acetic acid (Ethylic acid)	30.02	670
	Propanoic acid (Propionic acid)	0.72	699
	Capric acid (Decanoic acid)	21.81	1837
	Total	52.55	
2. Aldehydes	3-Methylbutanal	1.02	682
	2-Methylbutanal	1.41	685
	Hexanal (n-Hexanal)	1.74	779
	Benzaldehyde	1.61	1005
	Benzeneacetaldehyde (Hyacinthin)	1.18	1153
	Hex-2-enal, 2-isopropyl-, 5-methyl	1.46	1297
	5-Methyl-2-isopropyl-2-hexenal	0.70	1319
	5-Methyl-2-phenyl-2-hexenal	0.79	2126
	Total	9.91	
3. Ketones	6-Methyl-3,5-heptadien-2-one	1.02	850
	2-Heptanone	1.73	903
	1-Octen-3-one (Vinyl amyl ketone)	0.78	1040
	(Z)-6-Octen-2-one	0.73	1136
	Total	4.26	
4. Alcohols	1-Octen-3-ol	10.00	1043
	3-Octanol	4.94	1070
	Total	14.94	
5. Lactones	Dihydro-2(3H)-furanone (Butyrolactone)	3.17	930
	4-(1-Hydroxyethyl)-γ-butanolactone	0.95	1496
	2-Methyl-tetrahydrofuran-3-one	0.69	786
	Total	4.81	
6. Furans	2-Amylfuran	2.00	1062
	Total	2.00	
7. Nitrogen-Containing Heterocyclic Compounds	Methylpyrazine	1.02	805
	2-Acetylpyrrole	3.81	1196
	Total	4.83	
8. Terpenes	L-Limonene	3.92	1125
	Total	3.92	
9. Alkanes	Tetradecane	1.20	1892
	Total	1.20	
10. Aromatic/Polycyclic Compounds	6-Methoxy-2-(1-buten-3-yl)-naphthalene	1.58	2640
	Total	1.58	

## Data Availability

The original contributions presented in this study are included in the article/Supplementary Materials. Further inquiries can be directed to the corresponding author.

## Supplementary Materials

The following supporting information can be downloaded at: https://www.mdpi.com/article/10.3390/nu18061008/s1, Figure S1: Spores of *Lactarius deliciosus* used in the study; Figure S2: HPLC chromatogram of 80 ppm standards of phenolic compounds examined in the study; Table S1: Primers and their sequences used in the study.

## Abbreviations

The following abbreviations are used in this manuscript:

*L. deliciosus*	*Lactarius deliciosus* (L. ex Fr.) Gray
NADPH	Nikotinamid Adenin Dinükleotid Fosfat
FRAP	Ferric reducing antioxidant power
CUPRAC	Cupric reducing antioxidant capacity
ABTS	2,2′-azino-bis(3ethylbenzothiazoline-6-sulfonic acid
DPPH	2,2-diphenyl-1-picrylhydrazyl
HPLC	High-performance liquid chromatography
GC–MS	Gas chromatography–mass spectrometry
MTT	[3-(4,5-dimethylthiazol-2-yl)-2,5-diphenyltetrazolium bromide]
PCR	Polymerase chain reaction
DMEM	Dulbecco’s modified eagle medium
EDTA	Ethylenediaminetetraacetic acid
DMSO	Dimethyl sulfoxide
LDG-A	*L. deliciosus* polysaccharide
ATCC	American type culture collection
