# Supplementary material for: From Phytochemical Characterization to Energy Metabolism-Driven Molecular Responses: The Anticancer Potential of Lactarius deliciosus (L.) Gray in Breast Cancer Cells
Source: Nutrients. 2026 Mar 23;18(6):1008. doi: 10.3390/nu18061008 (PMC13029253; doi:10.3390/nu18061008)
Supplement: Supplementary file 1 [file nutrients-18-01008-s001.zip › nutrients-4192882-supplementary.pdf]

From Phytochemical Characterization to Energy Metabolism-Driven Molecular Responses: The Anticancer Potential of *Lactarius deliciosus (L.) Gray* in Breast Cancer Cells

Supplementary Materials

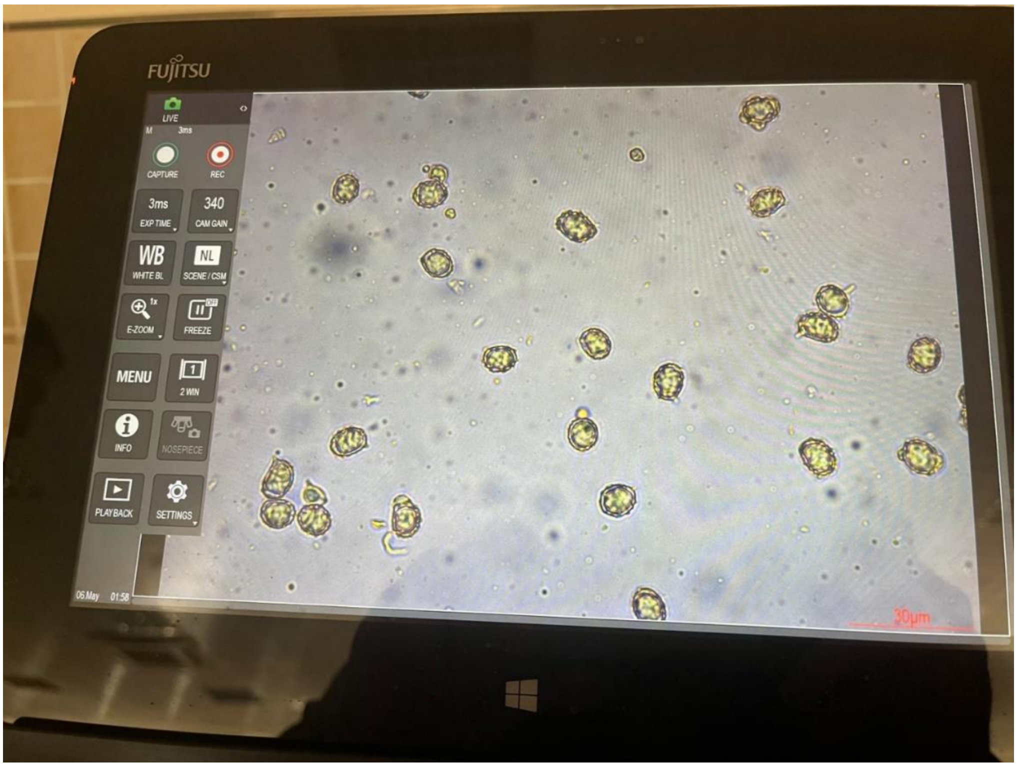

Figure S1. Spores of *Lactarius deliciosus* used in the study. The identification was utilized by Prof. Dr. Fuat BOZOK. A voucher number of L.Gülüm-1001 was given to the material and gat under protection in the biochemsitry laboratory of Horticulture Department of Bolu Abant İzzet Baysal University.

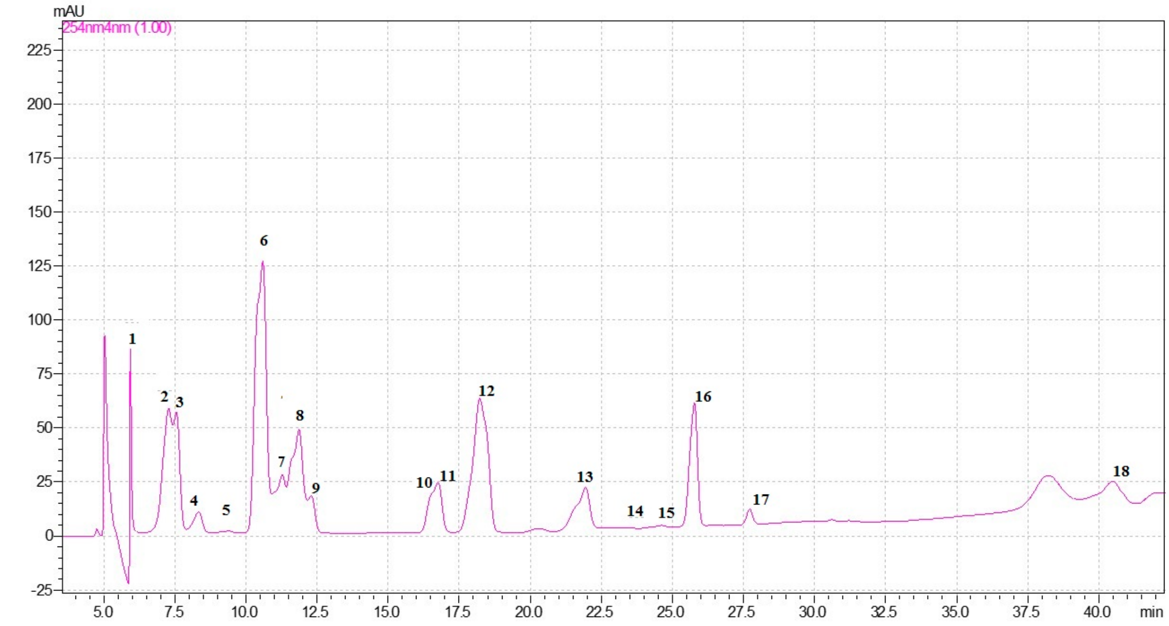

Figure S2. HPLC chromatogram of 80 ppm standards of phenolic compounds examined in the study. 1)gallic acid, 2) 4-aminobenzoic acid, 3)pro catechin, 4)chlorogenic acid, 5)syringic acid, 6)4-hydroxybenzoic acid, 7)syringin hydrate, 8)caffeic acid, 9)vanillic acid, 10)ferulic acid, 11)synapic acid, 12)coumaric acid, 13)rutintrihydrate, 14)quercitrin, 15)(-)-epicatechin, 16)(+)-catechin, 17)salicylic acid, 18)succinic acid.

Table S1. Primers and their sequences used in the study.

**Primer**	**Forward (5′-3′)**	**Reverse (5′-3′)**
GAPDH	TCGGAGTCAACGGATTTG	CAACAATATCCACTTTACCAGAG
ACTINB	GACGACATGGAGAAAATCTG	ATGATCTGGGTCATCTTCTC
ACLY	ACCTCAAATTTCATTGTGGG	GAAGATGAAGAACAGAACCAG
ACO2	AGAAGATTAGACTTGGACTCTC	GTGACCTTATCTTCTGTCATTG
ACSL1	AATGCGATCAACTACTATGC	CGTTACATATTGGGAGTGAG
ACSL3	CTAAACTAGACGAGAGTTTCC	CATCATAACCACAGGTCATC
ACSL4	ACCCGTATCATTTATGGAGG	TGTTTGGCATTGATGATGTC
ACSS3	GGGACCTGTCCTATATCAAG	TGAGGTAATATACGATGCGG
ADCY3	TGAAACTTTGTGCTGGAATC	GCTCCATTATCCCAATAGAC
CPT1C	CTTGTTCAGGTCTCTTATGC	TGCAGTCCTCTGATAAATTG
DHFR	CGTAAAGAAGAATTATCCGAGG	GTTTTCTAGACGTCCATTCAC
DNMT1	TTCCAGAAGTCAGATGATGC	CTTCCTTATAAACAGAACTGCC
ENO1	CACATTTGATGAGCTTGTTG	ATGAGTAAGATATCAACCCCG
FH	GGTCTTTCTTGAGGAAATCTG	AATTTTCCTCGTTCTCCAG
G6PD	GATATAAATGCAACAGAGGAGC	AGTTATCTTGTATGGCTTGC
GNPNAT1	TGAGTCTAGGACAGATTGTTG	AACATCTTCTACTCTTCCTCTC
GOT1	CTACGGCAGAGAAGAGTAAG	GCCATACTTGGAAGAAACTG
HK2	GGTTATTCTATGTTGCGTCTC	AATAACAGCTGGTAGAGGAG
HMGCS1	GATCCACGAGACCAACC	CAGGGTGAATCTTGTTGTAG
IDH1	GGAACATTACCTTGTCCTTG	CATGACCTTTTTCCCTTCAG
LDHAL6A	AGCATGCAGAAGTCAATG	ATTTTCCCACAACCTTCTTG
MAT2A	CCTCATATAGGGTATGACAAGG	GTAAATCACTTTGGACCCAG
ME1	TCTGTCCATCAAGATGAGTG	TACATGTAGCTGAAGAGTCG
MTR	ATTGAGAGCCAGATACAGAC	TAGCTATCTTGTCACCAACTC
OGDH	AGACAATTTGTATGCAGAGC	AATGGAGGTCACTCTCTTAG
PAFAH1B2	AAGGTCAAGGTGTTGAAATG	TCTCATTCTCCACATAGAGG
PDE4D	GTAAAGATCATCACCATCCTG	CCAGGATCAAGGAGATATGG
PDK2	CAAGAACACACCTGTCTATG	TTCTCCAGATCATTCAGGAAG
PFKFB4	GTTCTTGCCAATCCTGAATC	TGTCGACCTCTGTCTTATTC
PGAM1	AGTGTAGCTTACGACTGTG	CATAAGCTTTTGCAATTGGG
PGM2	CAATATGAGATATGGCGAGAG	TCTCCATTCTTTAGTCTCCAC
PHGDH	CAGAGGAGGTACTACAAACC	ATAAGGACGATATCCGAAGAG
PHGDM	TGAGTGGGTGATTATTGAAC	GTTGACTATGTACGTGATGG
PRPS1	TTGGCTTCATGATCTTTCAC	AATTTAACATCCCCAAAGGC
PSPH	GGTTCATTGGACACTATCTTG	GTTACATGCTCATGTGTTCC
SCD	AACCTCATAGCAAACCATTG	TCCTAGTTCTGGAATCCTTTC
SDHA	CACACAACAACTTCAACTTC	CATACAGTGTTTTCCAGTCC
SLC1A5	AAAAGCACAGAGTTGATGAG	GAGTGTCTTTGGCATACTTG
SLC2A2	ACAACCAGTCCTTCAATAAC	CTACATTGACTGTATTGCCC
SLC2A7	AGATAAGCAACATGAAGTGG	CAAGGACATCTTCTTGTTCAG
SLCR7A11	ATAAAGCAGAGTACCCTGAAG	CAAGTTTTCTGGGTTAGATCC
TK1	GGCATCTTGGTTATGAACAC	CTGTTCCCATGAACTTCTTC
TKTL1	TTACCACAACCTGAAGAATG	TCCAGGCCTTCTTTATTCTC
TPI1	CTACAGCAACATCAGTAAGG	GGGAACTATTTCTTCATTCCAG
UCKL1	CTAAGTAAGGGCCTCTATGG	CTTTAAAGCTTGAGCTCCTTC
